# Digital learning resource use among Swedish medical students: insights from a nationwide survey

**DOI:** 10.1186/s12909-025-07446-7

**Published:** 2025-06-11

**Authors:** Martin F. Bjurström, Emma Lundkvist, Louise W. Sturesson, Ola Borgquist, Robin Lundén, Malin Jonsson Fagerlund, Miklós Lipcsey, Thomas Kander

**Affiliations:** 1 Department of Surgical Sciences, Clinical Pain Research, Uppsala University, Uppsala, Sweden; 2 Department of Pharmacy, Clinical Pharmacy and Pharmacotherapy and Unit for Academic Teaching and Learning, Uppsala University, Uppsala, Sweden; 3 Department of Clinical Sciences Lund, Anesthesiology and Intensive Care Medicine, Lund University, Lund, Sweden; 4 Department of Anesthesiology and Intensive Care, Skåne University Hospital, Lund, Sweden; 5 Department of Physiology and Pharmacology, Section for Anesthesiology and Intensive Care Medicine, Karolinska Institute, Stockholm, Sweden; 6 Department of Surgical Sciences, Anesthesiology and Intensive Care, Uppsala University, Uppsala, Sweden; 7 Multidisciplinary Pain Center / Department of Anesthesiology and Intensive Care, Uppsala University Hospital; Department of Surgical Sciences, Uppsala University Hospital, Ingång 79, Våning 2, Uppsala, 751 85 Sweden

**Keywords:** Medical students, Education, Digital, Flashcards, Video, AI

## Abstract

**Background:**

Medical students navigate a complex landscape of digital tools with potential to enhance learning. The main objectives of the current study were to investigate which digital resources are being used, which background factors are associated with utilization, perceived advantages and disadvantages of different digital resources, and explore future directions.

**Methods:**

Cross-sectional, nationwide, online 25-item multiple-choice question survey and one free-text question enabling qualitative data analysis. Medical students at all seven universities with medical school programs in Sweden were invited to participate. Data were collected October – December 2024.

**Results:**

One thousand seven hundred sixty-six students responded to the survey, with an average response rate of 20.2% across sites. The five most frequently used digital resources were (percentage using at least on a weekly basis): University study platform (75.3%), videos (68.0%), flashcards (66.4%), student notes (53.4%) and external study platforms (47.3%). Flashcards were perceived to have a large to very large positive impact on development and maintenance of theoretical knowledge by 63.7% of students. Younger age (≤ 25 years) was strongly associated with higher use of flashcards (OR 1.98 (95% CI 1.54–2.54)) and generative artificial intelligence (AI) (OR 1.66 (1.29–2.15)), whereas having children at home was associated with more frequent use of videos (OR 2.32 (1.32–4.08)) and university digital platforms (OR 2.62 (1.26–5.45)), in multivariable logistic regression analyses. Most students (74.8%) reported finding their digital resources based on recommendations from more senior medical students. Perceived key advantages of digital resources in general were availability (90.9%), flexibility (80.6%), and more effective learning compared to traditional modalities (59.0%), while possible disadvantages included risk for distraction (49.6%) and uncertainty regarding reliability of content (45.4%). Qualitative data highlighted several areas of interest, including calls for universities and lecturers to provide high-quality, updated video material and flashcard decks tailored to the curriculum, and review and recommend third-party digital resources (e.g., YouTube channels).

**Conclusions:**

Medical students extensively use digital resources, with perceived large positive learning effects and benefits. Several background factors influence usage patterns. These data may support institutions, program directors and teachers in their efforts to guide and improve use of digital learning tools in medical schools.

**Supplementary Information:**

The online version contains supplementary material available at 10.1186/s12909-025-07446-7.

## Introduction

Technological advancements and digital resources influence and shape all aspects of modern society. Indeed, medical students today face a vast, rapidly transforming landscape of tools, with the potential to support and supplement parts of the medical education curriculum. Over time it appears that reliance on traditional learning resources, such as lectures and use of printed textbooks, have decreased [1], in favor of non-traditional asynchronous learning modalities, e.g., spaced repetition tools, educational videos, digital study platforms and webpages. Extensive and frequent use of these resources has been demonstrated in several small, single-institution surveys conducted in countries with different educational systems (e.g., Australia, Canada, Saudi Arabia, Sweden, United Arab Emirates, USA) [1–8]. It even seems as though third-party digital resources (i.e., e-resources not created by the medical school) may sometimes be the primary source of learning, during certain phases of medical school, with accelerated usage prior to examinations [1, 8]. These reported usage patterns may represent a strategic approach to learning, indicating that digital resources align better with medical students´ learning preferences and priorities than traditional methods. Nevertheless, group seminars and face-to-face lectures remain popular [7], and provide opportunities for real-life interaction with fellow students and faculty which cannot easily be replaced by digital methods.

Most currently enrolled medical students have grown up in a technology-rich environment and context where the use of digital resources in all dimensions of life is completely natural. This stands in sharp contrast to many senior faculty members and lecturers who often come from generations with more limited digital navigation skills. Hence, there is an apparent risk for mismatch in what tools medical students prefer and desire, and what is provided in form of faculty-developed resources. Given the amounts of facts and knowledge that medical students are expected to learn during often very brief courses and clinical rotations, it is not surprising that many turn to digital resources that can highlight the most important content, motivate and facilitate memorization, while helping to manage time, and perhaps even contribute to a better study – life balance. However, a key issue to examine is whether students choose strategies that enhance deep learning and understanding or focus on quickly acquiring a knowledge level necessary to pass exams. Importantly, students´ perceptions of learning are not necessarily correlated with their actual learning. Indeed, the feeling of learning might unintentionally prioritize inferior passive teaching methods over research-based pedagogical approaches [9].

This nationwide survey of medical students at all seven medical school programs in Sweden aimed to investigate and evaluate (1) which digital resources are being used, (2) which background factors are associated with use of different digital resources, (3) usage patterns, e.g., dynamic changes over the course of the medical programs, and (4) perceived advantages and disadvantages of different digital resources. Based on comprehensive survey study methodology and pilot-testing, digital resources of interest included: generative AI, digital flashcards, university study platforms, external study platforms, social media groups, digital material from senior medical students, e-books, digital articles, podcasts and educational videos, the latter including both lectures recorded by the institution and videos from third-party resources.

The overarching objective of the study was to achieve a better understanding of medical students´ learning methods and techniques, to support and enable future recommendations, tailoring and design of digital learning resources. In contrast to previous single-institution attempts to investigate use of digital resources among medical students, our study was nationwide, with the potential to generate more broadly generalisable results.

## Methods

### Overview of study design

This cross-sectional, nationwide survey study aimed to evaluate the use of digital resources among medical students in Sweden. Students from all seven universities with medical school programs were invited to participate, i.e., (ordered geographically from south to north) Lund, Göteborg, Linköping, Örebro, Stockholm, Uppsala, and Umeå. Since no sensitive personal data were collected, the Swedish Ethical Review Authority presented no ethical objections to the project, deemed the study exempt from full review, and waived the need for consent to participate (Dnr 2024–03072-01). The manuscript is reported according to the Checklist for Reporting of Survey Studies (CROSS) (Supplemental Checklist).

### A brief overview of medical school programs in Sweden

Although there are some minor differences in the structure of semesters across medical schools in Sweden, the program currently consists of 11 semesters (5.5 years), with the following basic framework: semesters 1–4 basic science (often referred to as ´preclinical´), 5–6 clinical theory and introduction to clinical work, 7–9 [10] clinical theory and practical clinical work, (10)−11 more advanced clinical work. A master´s thesis is completed within the scope of the program. Semesters 1–6 are generally considered as basic/fundamental level, while semesters 7–11 are termed advanced.

### Process of survey creation

The survey study group (authors) consisted of senior consultants with central roles and responsibility for teaching medical students (anesthesiology, pain medicine), and one pedagogical expert (EL). To ensure comprehensive coverage of digital learning tools, the selection of resource types to be included in the questionnaire was based on a combination of:

1. the authors´ collective experience as medical educators
2. a targeted literature review of similar national and international studies
3. in-depth interviews with 10 medical students from two different universities (Lund and Uppsala), and
4. iterative pilot-testing within the same student group.

A first draft version of the survey included 25 questions. Based on extensive comments and feedback, the survey was amended as follows: addition of response alternatives (question 5, 6, 13, 23), rephrasing of questions (question 7, 16, 20). Upon completion of these edits, further pilot-testing and iterative refinement resulted in the following changes: rephrasing of response alternatives (question 5, 6, 7), rephrasing of questions (question 15, 23), addition of one response alternative (question 13, 15), reordering of some questions, and addition of a new question (question 26, a free-text response question enabling qualitative data collection). The final version of the 26-question survey was approved by all group members (Supplemental Document 1 (translated English version), Supplemental Document 2 (original Swedish version)). Briefly, the domains covered were basic information (questions 1–4, 23–25), digital resources in general (questions 5–12), video (questions 13–15), podcasts (question 16), flashcards (questions 17–18), generative AI (questions 19–20), course literature (questions 21–22). Only the Swedish version of the survey was used throughout the study.

### Enrolment and data collection

Prior to enrolment, approval was obtained from all medical school program directors at the seven universities. The REDCap electronic data capture tool hosted at Lund University, was used for data collection and management [10, 11]. E-mail addresses to currently active medical students were obtained from the administrative department at each university, whereafter all medical students were e-mailed an invitation to take part in the study, including URL and QR-code link to the REDCap survey. Three reminders were e-mailed over the course of the predetermined data collection period (07–10–2024 – 22–12–2024). Participation was completely voluntary, with no economic or other incentive to take part. To enhance enrollment, two medical students at each site were invited to assist with local study enrolment through social media channels and fliers distributed throughout key places at their respective campuses. The twelve students who assisted with enrolment were each given a gift card worth 1000 SEK. Additional methods to potentially increase enrolment were brief information about the study during lectures given by the authors (Uppsala) as well as e-mail notification about the study to the director of studies for the medical school programs. The recruitment method aimed to target a random, representative sample of Swedish medical students. No personally identifiable information was collected.

### Statistical methods

Descriptive statistics were used to summarize data, mainly as absolute numbers and percentages of respondents. The effects of different categorical background factors (age ≤ 25 vs. > 25 years, sex [female vs. male], having children at home vs. no children or no children at home, extracurricular work on a weekly basis vs. occasionally or no additional work, preclinical stage vs. clinical semesters) on frequency of use of digital resources were examined through Chi-square statistics, as well as multivariable logistic regression models. The Hosmer–Lemeshow goodness of fit test was valid for all logistic regression models (*p*-values > 0.05). No variables used in the analyses were modified. Only data from participants who completed the full survey were used, hence there were no missing data. Sample size was determined by the preplanned enrolment period, no power calculations were performed. A *P*-value < 0.05 was considered significant. Data management and analyses were conducted utilizing SPSS version 29.0 (Armonk, NY: IBM Corp.).

## Results

### Survey participants and response rate

Basic characteristics of survey respondents are provided in Table 1.

**Table 1:** Basic characteristics of survey respondents (*n* = 1766)

University and demographic characteristics	N	%
University		
Lund	383	21.7
Göteborg	181	10.2
Linköping	242	13.7
Örebro	191	10.8
Stockholm	213	12.1
Uppsala	311	17.6
Umeå	245	13.9
Age (years)		
< 20	82	4.6
20–25	1228	69.5
26–30	274	15.5
31—35	96	5.4
36–40	47	2.7
> 40	39	2.2
Sex		
Female	1126	63.8
Male	625	35.4
Other	7	0.4
Does not wish to report	8	0.5
Previous work/study experience		
University, some courses	494	28.0
University, full program	226	12.8
Vocational education	32	1.8
Residential college for adult education	90	5.1
Military service	131	7.4
Work experience < 5 years	650	36.8
Work experience ≥ 5 years	253	14.3
None	526	29.8
Extracurricular work during semesters		
Occasionally	522	29.6
5–10 h/week	196	11.1
11–20 h/week	61	3.5
> 20 h/week	26	1.5
None	961	54.4
Children		
Yes, living at home	107	6.1
Yes, not living at home	11	0.6
No	1644	93.1
Does not wish to report	4	0.2

The response rate varied significantly between universities (12.3% – 28.0%; Lund 383/1370 = 28.0%, Göteborg 181/1474 = 12.3%, Linköping 242/1187 = 20.4%, Örebro 191/689 = 27.7%, Stockholm 213/1645 = 12.9%, Uppsala 311/1121 = 27.7%, Umeå 245/1236 = 19.8%). Total response rate across all seven sites was 1766/8722 = 20.2%. The sample was representative according to sex-distribution for the six out of seven sites that provided pertinent data (% females [% females at site]): Lund 62.4% [58.8%], Göteborg 60.2% [not reported], Linköping 62.8% [60.2%], Örebro 66.0% [65.0%], Stockholm 55.9% [54.5%], Uppsala 70.1% [64.6%], Umeå 66.5% [60.0%].

Student distribution across semesters can be found in Supplemental Figure 1. A majority of students attended clinical semesters, i.e., semesters 5–11 (*n* = 1035, 58.6%), whereas 731 attended preclinical semesters, i.e., semester 1–4 (41.4%).

Approximately four out of five study participants (*n* = 1426, 80.7%) responded to the final free-text answer question, providing vast qualitative data, ranging from three words to 360 words.

### Which digital resources are used?

**Fig. 1:**
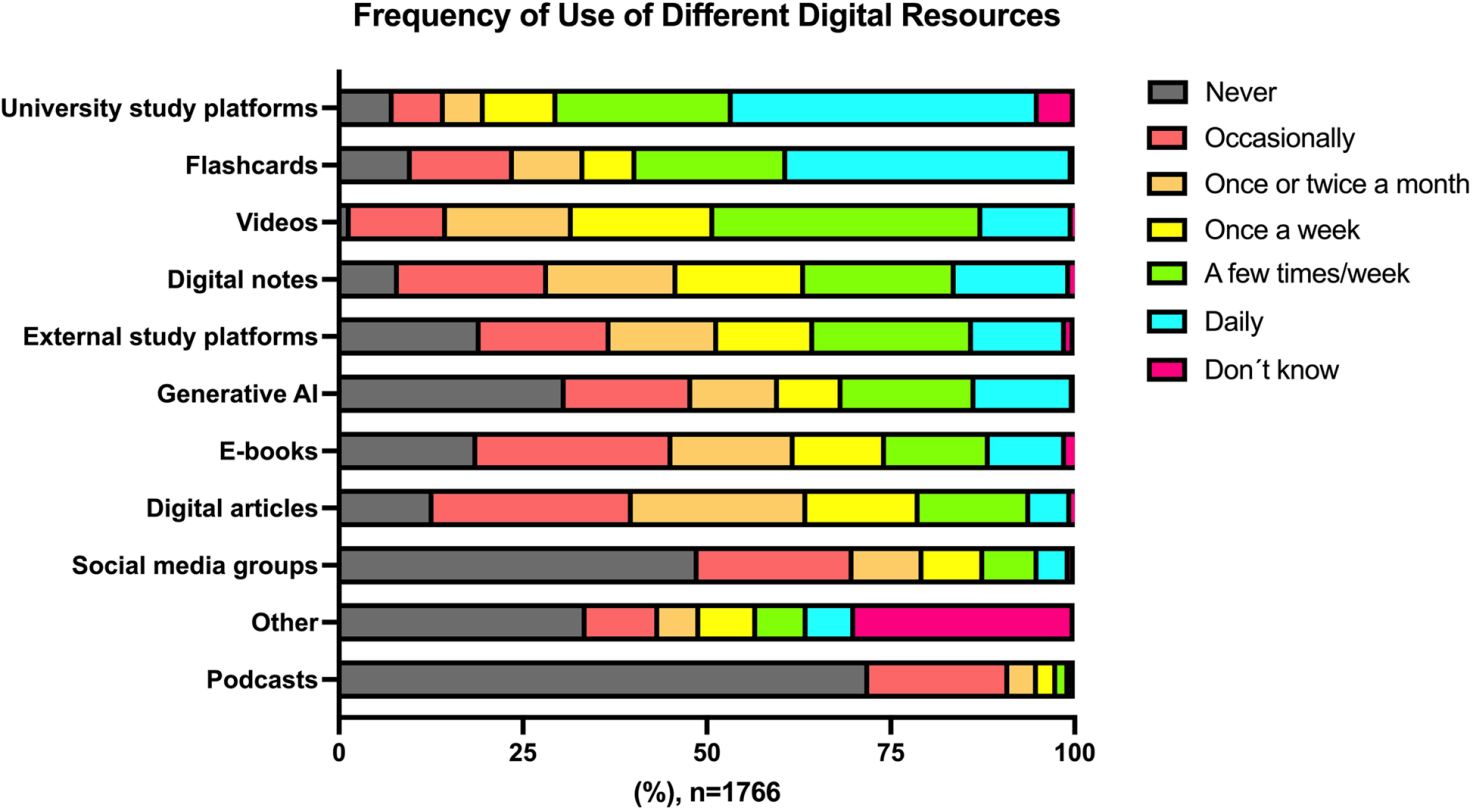
Frequency of use of different digital resources

Frequency of use of different digital resources is shown in Fig. 1. The five most used digital resources were (percentage at least on a weekly basis): university digital study platform (75.3%), video (68.0%), digital flashcards (66.4%) digital student notes (53.4%), and external digital study platform (47.3%). Differences in the use of digital resources between the seven universities can be seen in Supplemental Figure 2. Usage frequency of several modalities varied substantially between universities, for example, at least weekly use of video (55.0% [Uppsala] – 81.9% [Umeå]), flashcards (51.3% [Örebro] – 84.0% [Lund]) and generative AI (29.8% [Lund] – 50.4% [Linköping]).

Qualitative data (free text format responses to the final survey question) highlighted frequent use of multiple different specific digital resources including flashcard programs (e.g., Anki, Quizlet), generative AI (e.g., Chat GPT), YouTube channels (e.g., Ninja Nerd), external study platforms (e.g., Hypocampus, Osmosis), webpages (e.g., internetmedicin.se, Wikipedia), databases and search engines (e.g., PubMed, ClinicalKey, Google), and workspace and notebook applications (e.g., OneNote, Notion, Trello). A complete list of reported digital resources can be found in Supplemental Table 1. An overview of thematic analysis of free-text responses is provided in Supplemental Table 2. Although select, illustrative quotes can be found in the text below, a full presentation of the qualitative findings will be provided in a separate, Swedish-language article.

### Which background factors influence frequency of use of digital resources?

**Fig. 2:**
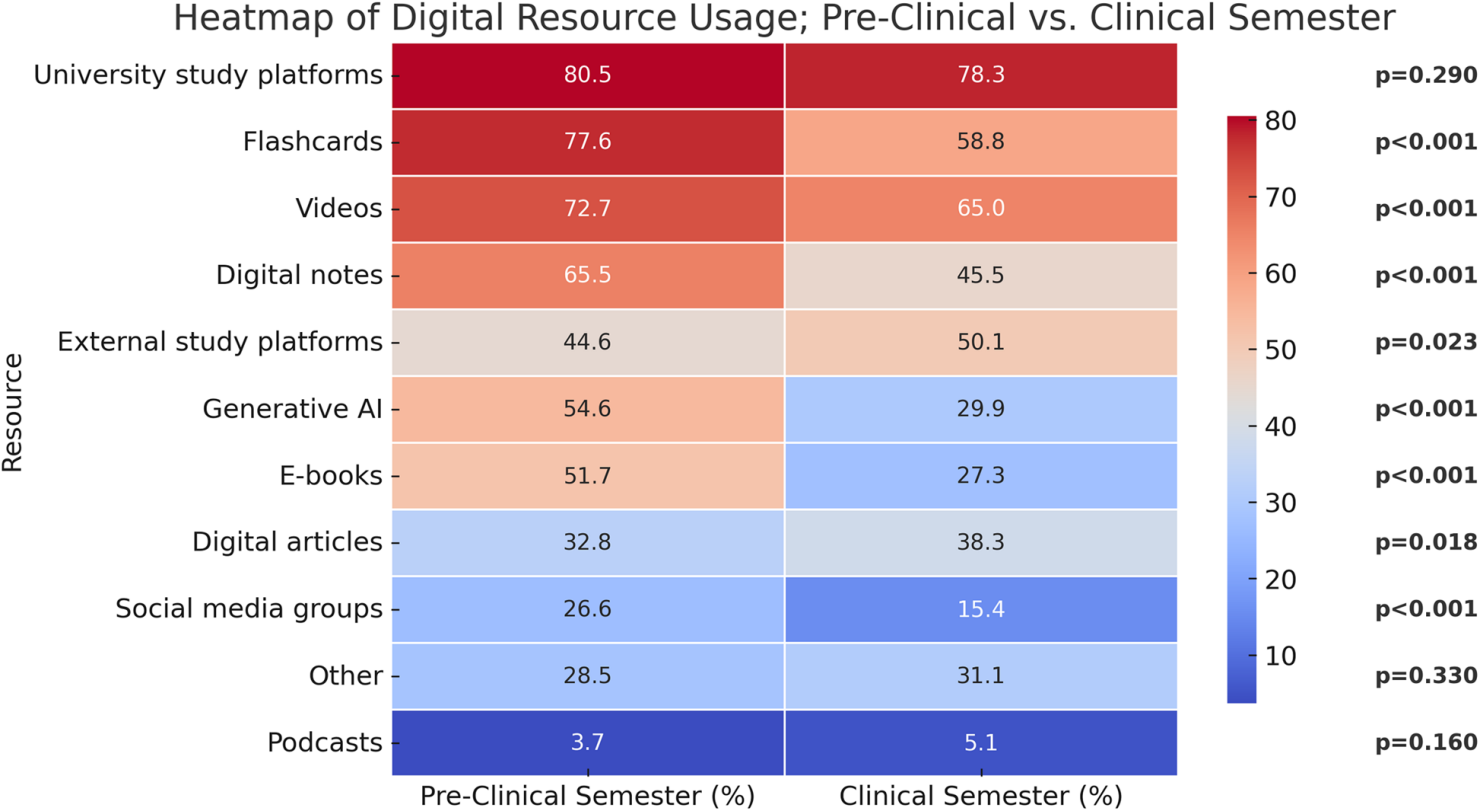
Digital resource use for those attending preclinical vs. clinical semesters

Subgroup analyses revealed significant differences in the frequency of use of digital resources stratified according to age, sex, having children, regular extracurricular work, and stage of medical program (Table 2). Most differences were found when comparing resource use for those attending preclinical vs. clinical semesters, with preclinical students reporting significantly higher use of videos, flashcards, social media groups, generative AI, student notes, and digital books (Table 2, Fig. 2). In multivariable logistic regression analyses, younger age (≤ 25 years) was strongly associated with more frequent use of flashcards and generative AI, but more infrequent use of digital articles and podcasts (Table 3), although very few overall reported using podcasts in relation to medical school (Table 2). Female sex was associated with less frequent use of generative AI and digital articles, but higher use of digital student notes and external digital platforms (Table 3). Having children at home was associated with more frequent use of videos and university digital platforms (Table 3).

**Table 2:** Frequency of use of study resources stratified according to key background factors

Weekly use^α^	Age ≤ 25 vs. > 25	Female vs. male	Children vs. no children^β^	Extracurricular work vs. no work	Preclinical vs. clinical
Video	875/1307 (66.9%) vs. 325/454 (71.6%)	764/1122 (68.1%) vs 428/624 (68.6%)	86/105 (**81.9%**) vs 1111/1652 (**67.3%**)**	194/281 (69.0%) vs 1006/1480 (68.0%)	529/728 (**72.7%**) vs 671/1033 (**65.0%**)***
Podcasts	40/1303 (**3.1%)** vs 40/455 (**8.8%**)***	48/1119 (4.3%) vs 32/624 (5.1%)	14/106 (**13.2%**) vs 66/1648 (**4.0%**)***	25/282 (**8.9%**) vs 55/1476 (**3.7%**)***	27/726 (3.7%) vs 53/1032 (5.1%)
Flashcards	931/1306 (**71.3%**) vs. 241/455 (**53.0%**)***	746/1122 (66.5%) vs 419/624 (67.1%)	49/106 (**46.2%**) vs 1120/1651 (**67.8%**)***	168/282 (**59.6%**) vs 1004/1479 (**67.9%**)**	564/727 (**77.6%**) vs 608/1034 (**58.8%**)***
Social media groups	272/1299 (20.9%) vs 79/455 (17.4%)	230/1117 (20.6%) vs 119/622 (19.1%)	24/107 (22.4%) vs 327/1643 (19.9%)	52/281 (18.5%) vs 299/1473 (20.3%)	193/725 (**26.6%**) vs 158/1029 (**15.4%**)***
Generative AI	558/1308 (**42.7%**) vs. 150/456 (**32.9%**)***	420/1126 (**37.3%**) vs 283/623 (**45.4%**)***	37/107 (34.6%) vs 669/1653 (40.5%)	119/283 (42.0%) vs 589/1481 (39.8%)	399/731 (**54.6%**) vs 309/1033 (**29.9%**)***
Student notes	723/1303 (**55.5%**) vs. 220/452 (**48.7%**)*	629/1120 (**56.2%**) vs 311/620 (**50.2%**)*	45/106 (**42.5%**) vs 898/1645 (**54.6%**)*	147/281 (52.3%) vs 796/1474 (54.0%)	473/722 (**65.5%**) vs 470/1033 (**45.5%**)***
Univ. digital platform	963/1234 (78.0%) vs. 367/446 (82.3%)	856/1065 (80.4%) vs 462/600 (77.0%)	95/104 (**91.3%**) vs 1233/1572 (**78.4%**)**	228/272 (**83.8%**) vs 1102/1408 (**78.3%**)*	552/686 (80.5%) vs 778/994 (78.3%)
Ext. digital platform	603/1294 (46.6%) vs. 232/451 (51.4%)	557/1112 (**50.1%**) vs 272/618 (**44.0%**)*	62/105 (**59.0%**) vs 771/1636 (**47.1%**)*	127/281 (45.2%) vs 708/1464 (48.4%)	323/724 (**44.6%**) vs 512/1021 (**50.1%**)*
Digital books	501/1303 (38.4%) vs. 155/454 (34.1%)	408/1120 (36.4%) vs 240/622 (38.6%)	39/106 (36.8%) vs 616/1647 (37.4%)	86/283 (**30.4%**) vs 570/1474 (**38.7%**)**	374/724 (**51.7%**) vs 282/1033 (**27.3%**)***
Digital articles	431/1305 (**33.0%**) vs. 203/453 (**44.8%**)***	352/1121 (**31.4%**) vs 275/622 (**44.2%**)***	51/106 (**48.1%**) vs 582/1648 (**35.3%**)**	108/283 (38.2%) vs 526/1475 (35.7%)	238/725 (**32.8%**) vs 396/1033 (**38.3%**)*
Course books	498/1296 (**38.4%**) vs. 203/452 (**44.9%**)*	433/1112 (38.9%) vs 263/622 (42.3%)	58/105 (**55.2%**) vs 642/1640 (**39.1%**)***	110/283 (38.9%) vs 591/1465	291/713 (**40.8%**) vs 308/1021 (**30.2%**)***

^α^weekly use includes the following answers: one time/week, a few times/week, daily

^β^having children living at home vs. no children or no children living at home

^*^p < 0.05

^**^p < 0.01

^***^p < 0.001

Pearson Chi-square test (asymptotic significance, 2-sided); bold font indicates statistically significant group differences

Participant numbers differ between digital modalities given that “don´t know” responses are excluded from the analyses

**Table 3:** Multivariable logistic regression analyses: associations between background factors and weekly use of different digital resources

	Videos		Podcasts		Flashcards		Social media groups		Generative AI	
	OR (95% CI)	P	OR (95%CI)	P	OR (95%CI)	P	OR (95%CI)	P	OR (95%CI)	P
Younger age^α^	0.93 (0.71-1.20)	0.56	0.45 (0.27-0.75)	0.002	1.98 (1.54-2.54)	<0.001	1.35 (0.98-1.85)	0.07	1.66 (1.29-2.15)	<0.001
Sex: female	0.98 (0.80-1.22)	0.88	0.90 (0.56-1.43)	0.65	0.92 (0.74-1.14)	0.44	1.07 (0.84-1.37)	0.58	0.70 (0.57-0.85)	<0.001
Children^β^	2.32 (1.32-4.08)	0.003	1.97 (0.99-3.94)	0.05	0.69 (0.44-1.06)	0.09	1.51 (0.89-2.57)	0.13	1.15 (0.73-1.82)	0.54
Extracurricular work^γ^	1.00 (0.75-1.33)	0.99	1.94 (1.17-3.24)	0.011	0.82 (0.62-1.08)	0.15	0.94 (0.67-1.31)	0.70	1.21 (0.93-1.59)	0.16
	Student notes		Univ. digital platforms		Ext. digital platforms		Digital books		Digital articles	
	OR (95% CI)	P	OR (95%CI)	P	OR (95%CI)	P	OR (95%CI)	P	OR (95%CI)	P
Younger age	1.18 (0.92-1.50)	0.19	0.95 (0.70-1.29)	0.74	0.86 (0.67-1.09)	0.21	1.16 (0.90-1.50)	0.24	0.66 (0.51-0.84)	<0.001
Sex: female	1.26 (1.03-1.53)	0.02	1.22 (0.96-1.56)	0.11	1.29 (1.06-1.57)	0.01	0.89 (0.73-1.09)	0.27	0.59 (0.48-0.73)	<0.001
Children	0.71 (0.46-1.11)	0.13	2.62 (1.26-5.45)	0.01	1.48 (0.95-2.31)	0.09	1.14 (0.72-1.79)	0.59	1.26 (0.81-1.96)	0.30
Extracurricular work	0.99 (0.76-1.29)	0.96	1.37 (0.96-1.96)	0.08	0.85 (0.65-1.11)	0.23	0.70 (0.53-0.93)	0.01	0.96 (0.73-1.26)	0.77

^α^Age <=25 vs >25 years

^β^Children at home vs no children at home

^γ^Extracurricular work on a weekly basis vs occasionally/no work

*Abbreviations*: *CI* confidence interval, *OR* odds ratio

### How do medical students find their digital resources?

Most students (74.8%) reported finding their digital resources based on recommendations from more senior medical students, through online search engines (57.0%) or by recommendations from the course management (40.7%). Few (7.6%) indicated ‘other’ ways of finding resources, and only 4.8% reported selecting resources after watching ads while using other digital resources.

### Is use of digital resources promoted?

More than half of students reported that use of digital resources was promoted by the course management group (often, 19.6%; sometimes, 44.7%), whereas a significant minority responded ‘rarely’ (22.9%) or ‘never’ (5.4%). The extent to which such promotion influences use of digital resources appears to vary widely (high, 12.2%; moderate 37.2%; low 24.6%, not at all, 17.2%; don´t know 8.7%).

### In which situations and contexts do medical students most often use digital resources?

**Fig. 3:**
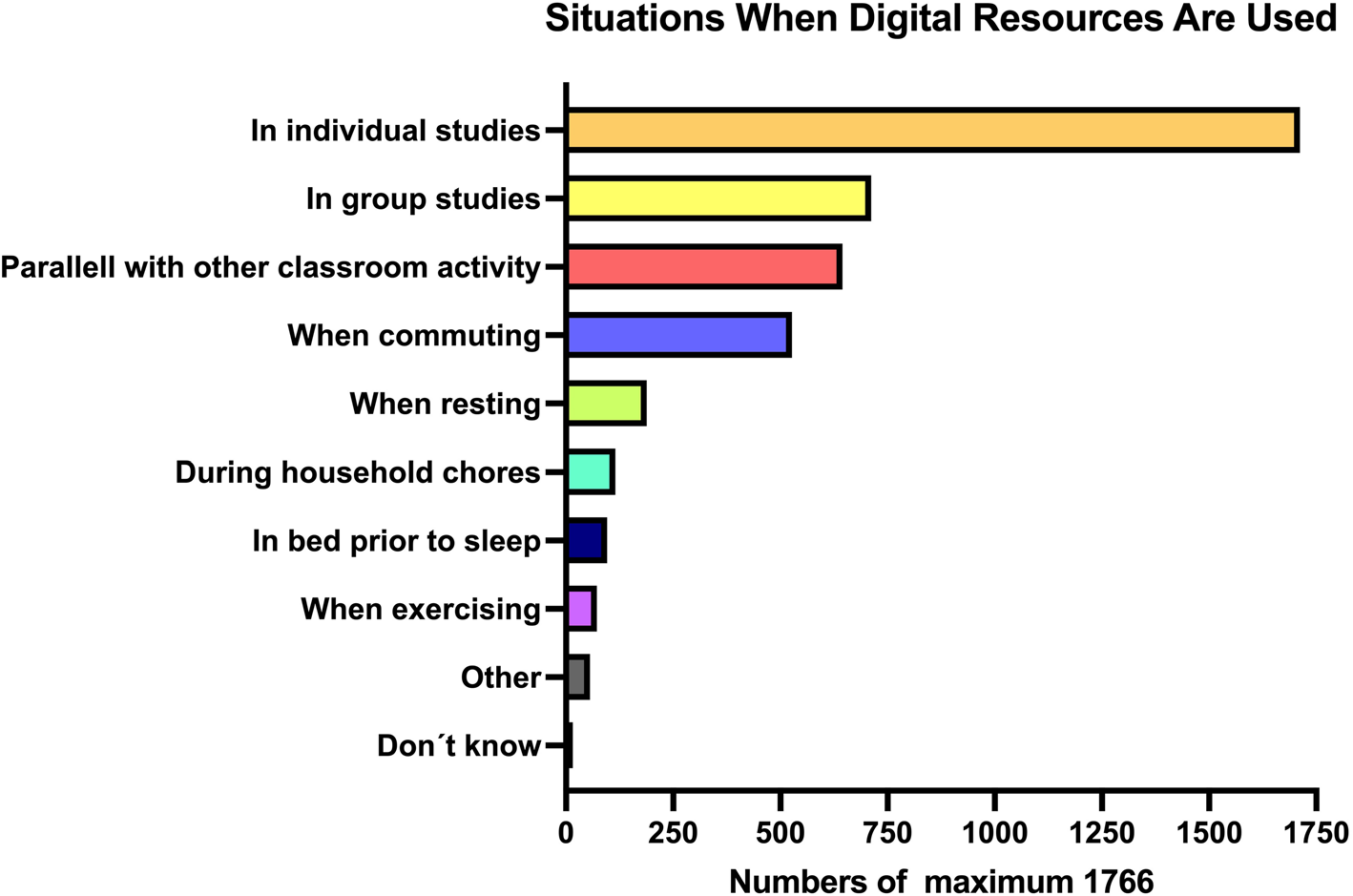
Situations and contexts in which digital resources are most frequently used by medical students

Results related to which situations and contexts digital resources are used in are shown in Fig. 3. Almost all medical students (96.9%) reported using digital resources during individual studies.

### Perceived advantages and disadvantages of digital learning resources in general

For digital resources in general, the reported primary advantages were availability (i.e., being able to decide when and where to use [90.9%]), possibility to pause/repeat [80.6%], more effective learning compared to other traditional learning methods [59.0%], updated/relevant content [49.0%], and ability to multi-task [17.3%]. The most common reported disadvantages were being easily distracted [49.6%], unreliable content [45.4%], difficulties separating study time and leisure time [40.5%], and difficulties finding useful content [21.6%]. Fewer than 10% reported that they found digital study resources less effective compared to other traditional methods [7.0%], or that the number of ads or commercial breaks was burdensome [8.7%]. Almost one out of five students were unable to identify any specific disadvantages associated with digital resources.

### Perceived advantages and disadvantages of educational videos

Perceptions related to educational videos were specifically probed. Similar to digital resources in general, the two most common advantages were the possibility to pause/replay [89.8%] and availability (being able to decide when and where to use [84.8%]). Moreover, more than half [55.0%] found videos to be an effective way to quickly acquire concentrated knowledge, as well as prepare for upcoming classroom activities/lectures [50.5%]. Forty-one percent reported that videos may add a dimension that is hard to capture with other learning modalities and 33.0% found videos to provide more effective learning compared to other traditional methods. Only 2.8% reported not using videos for learning. Although only 1.1% were unable to see any advantages of video-based learning, several disadvantages were reported: being easily distracted [44.1%], difficulties judging whether the video content is applicable in a Swedish context [43.4%], difficulties finding videos with the desired learning content [31.9%], subpar quality [29.3%], unreliable information [22.1%] and too many ads/commercial breaks [10.5%].

The main reasons for discontinuing a video prematurely were as follows: does not fulfill learning needs [54.1%], message not sufficiently focused [36.0%], too low tempo [33.0%], presenter/lecturer not engaging enough [24.3%], subpar sound quality [19.1%], the program does not set aside enough time to view the recommended video material [17.6%], content does not match the title [9.6%] and subpar image quality [6.6%].

Qualitative data provided by 46 participants highlighted a few areas that were not captured by the survey questions:

- content is sometimes outdated, especially the universities´ material
- students sometimes watch the videos at a playback speed > 1 × to optimize their use of limited study time
- the quality and content of professional YouTube videos (certain creators are mentioned several times, e.g., Ninja Nerd) are often perceived markedly better than the universities´ videos: “…you get access to the best lecturers on the planet.” (participant #257)
- some students wish that teachers/lecturers could recommend, evaluate YouTube channels
- disadvantage with prerecorded video lectures: not being able to ask questions in real-time
- being able to watch video from home, even if you are sick

### Perceived effect of simultaneous exercise on podcast-based learning

One question was devoted to exploring how medical students perceive the effects of exercise on learning while listening to podcasts. More than half of students [57.1%] checked the response option ‘I don´t listen to medical educational podcasts or don´t exercise’. For the remaining students, the perceived direction of the effect varied substantially: no effect [1.9%], very negative effect [4.9%], somewhat negative effect [7.8%], some positive effect [11.3%], very positive effect [3.7%], and 13.3% responding ‘don´t know’.

### Flashcard-based learning: perceived effects and access to material

**Fig. 4:**
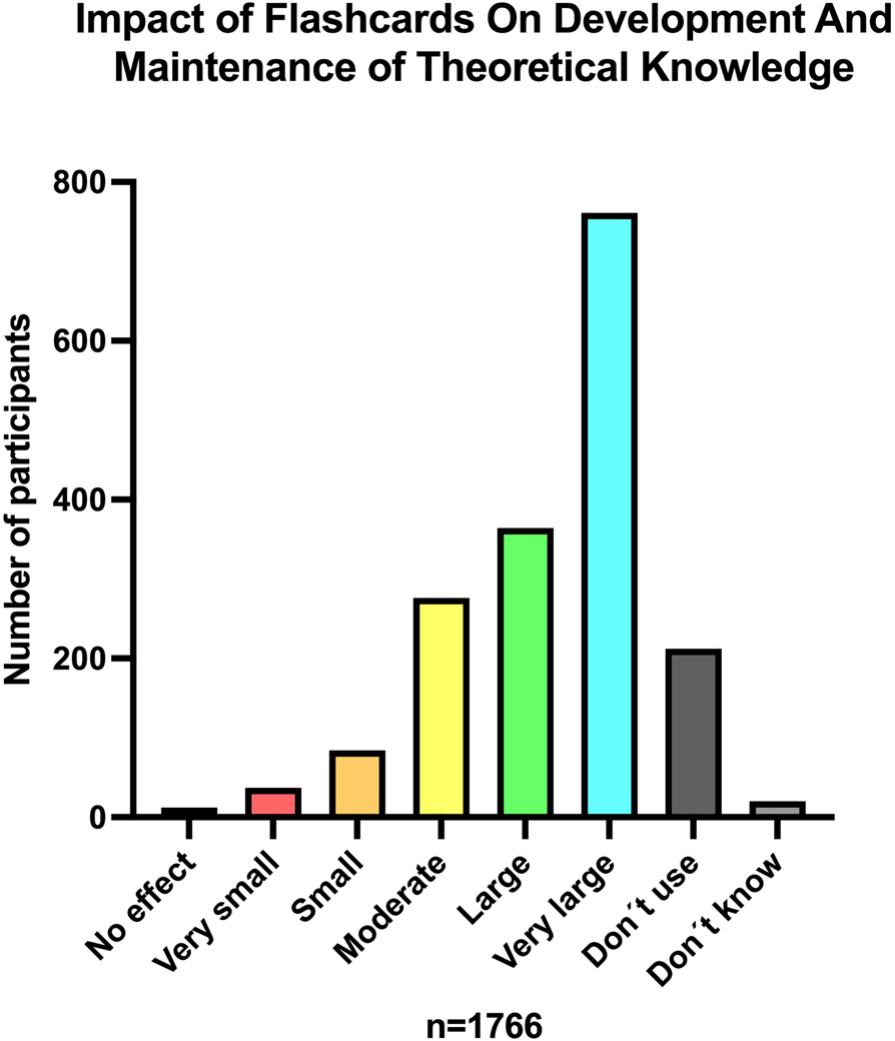
Perceived effects of flashcard-based learning on development and maintenance of theoretical knowledge

Flashcards were perceived to have a large to very large positive impact on development and maintenance of theoretical knowledge by 63.7% of medical students (Fig. 4).

There was an almost overwhelming praise of flashcards as a learning method, evidenced by extensive free-text answers from 32 participants, for example:

“I think Anki flashcards is the most effective way to learn. To create the cards you need to learn the subject, i.e., the creation itself becomes a learning process, and you can then repeatedly use the cards to learn.” (participant #54).

“Anki is the single most important factor in my studies.” (participant #946).

“Anki – cannot be overstated how much Anki helps medical students.” (participant #1064).

Several participants called for flashcards to be created by lecturers and course leaders, to accompany the educational material:

“It is a no-brainer that lecturers should hand out flashcards covering the content of the lecture, at least when we´re talking about anatomy and topics which demand intense memorization.” (participant #956).

“If I had one wish how digital resources could be used to improve medical school, it would be Anki-cards created and double-checked by the course leaders.” (participant #1731).

Flashcards were most commonly acquired from medical students attending higher semesters [65.7%], created by themselves [55.4%], or by fellow students attending the same course/semester [32.4%]. More rarely, flashcards were obtained from free online sources [21.5%] or generated de novo through specialized software, based on text/notes [5.9%].

### Generative AI: reasons for using and frequency of use related to submission tasks

The main reasons for using generative AI were to search for information or find answers to questions [51.8%], summarize text [28.4%], find ideas to get started with tasks [24.0%], adjust/improve text [17.3%], receive feedback on texts [14.3%], and translate text [12.0%]. Students reported using generative AI for submission tasks relatively infrequently: never [41.3%], rarely [23.0%], sometimes [17.8%], often [11.9%], always [4.1%]. However, younger students (age ≤ 25 years) reported using AI sometimes/often/always for submission tasks more often than those > 25 years old (35.9% vs 30.7%, χ^2^ = 3.9, *p* = 0.049).

Qualitative free-text data from 33 participants supported the wide utility of generative AI for medical students, and several students ranked generative AI (typically ChatGPT) as the single most useful digital resource.

### Course literature: printed books and digital books

Relatively few medical students reported using (buying or borrowing) traditional printed course literature for every course [16.6%] or most courses [23.0%]. Most students reported using printed course literature for a few courses [36.6%]. Approximately one out of five students [22.7%] reported never using printed course literature. The main reasons for not using course literature were as follows: more efficient learning using other methods [36.6%], expensive [26.4%], not needed to fulfill learning objectives [16.9%], not being able to borrow the book [3.7%], too difficult/complicated [3.2%].

The frequency of use of digital books (e-books) is shown in Fig. 1. Ample qualitative data from 30 participants highlighted several additional points related to course literature:

- free access to online e-books is appreciated (for example, via university subscription to ClinicalKey)
- many students find the course literature list “unrealistic” – sometimes several extensive books are included even for brief courses
- lack of time is commonly indicated as a reason for not using the designated course literature
- printed books may be heavy and cumbersome to carry around
- e-books are preferred by many given the possibility to search for keywords, more easily navigate the chapters and copy images/text to student notes

## Discussion

### Main findings

In this nationwide survey of 1766 medical students, we identified extensive use of multiple digital learning resources, such as study platforms, videos, flashcards and generative AI. Compared to their older peers, students < 25 years old reported using more flashcards, AI and student notes, whereas students having children at home reported more frequent use of videos, podcasts, study platforms, and digital articles. Perceived key advantages of digital resources in general were availability, flexibility, and more effective learning compared to traditional modalities, while possible disadvantages included risk for distraction and uncertainty regarding reliability of content, and difficulty separating study time and leisure time. Qualitative data highlighted several areas of interest, including calls for universities and lecturers to provide high-quality, updated video material or at least evaluate and recommend specific YouTube channels and medical education content creators. Additionally, several students called for lecturers and course leaders to create customized flashcard decks, to accompany lecture handouts. Given the high frequency of digital resource use, it is not surprising that fewer than four out of ten medical students reported using traditional printed course literature for most courses. These data are highly relevant for program directors and educators, with the potential to guide development and improvement of learning tools and allocation of resources in medical schools.

### Flashcard-based learning

As seen in our results, flashcards (typically two-sided, with the prompt on one side, and the information about the prompt on the other side; see Supplemental Figure 3 for an illustrative AI-generated example) is one of the digital resources most frequently used by Swedish medical students. One main pedagogical benefit of using flashcards is *spacing*, i.e., to separate study sessions in time, studying more continuously throughout the course instead of cramming before the exam. Spacing has been shown to increase student learning [12–14]. Several of the systems that students use are based on algorithms that maximize the effect of spacing. Another benefit of flashcards is *retrieval practice*, i.e. recalling facts or concepts from memory to enhance learning. Thus, taking a test will not only give feedback on what you know but can also enhance later retention, i.e., testing can improve learning [15, 16]. To maximize the benefits of flashcard-based learning, flashcards need to be used in a way that ensures the effects of both spacing and retrieval practice. Previous studies have shown that students often skip checking the answers, i.e., skip the self-testing, and that they do not always use flashcards with long enough lag-time between sessions, i.e., the full effect of spacing is not harnessed [17–19]. Since the questions in our survey did not cover *how* flashcards are used, we do not know if flashcards are used in a way that really enhances learning or not. In several free-text answers we notice requests for teachers to create flashcards; discussing how to best use those flashcards might be an even more important role for teachers.

The use of flashcards is especially correlated to subjects where you need to memorize detailed facts and terms. It is therefore not surprising that we see a significantly higher use during preclinical semesters where basic science, entailing the need to acquire detailed factual knowledge, usually plays a bigger role. Notably, more than six out of ten students perceived flashcards to have a large to very large positive impact on development and maintenance of theoretical knowledge. Might there be a risk that students perceive the effect to be so beneficial that they continue using flashcards on later courses where memorizing facts has a lesser role? Even during the clinical semesters, more than 50% report using flashcards on a weekly basis. Memorization may be important for some subjects, but understanding is also a vital part of learning and flashcards are not always used in a way that promotes understanding [20].

Loving et al. studied the use of flashcards in an anatomy course. Students found premade flashcards more useful than flashcards from a third party, perceiving flashcards from their peers as more relevant [21]. This corresponds to our results, where acquiring flashcards from more upper-level students was the most common strategy, followed by creating flashcards themselves. The latter has been shown to improve learning more compared to using premade cards [22].

### Generative AI

Our results indicate that generative AI has become an important tool for many medical students, aiding in summarizing information, creating study questions, simplifying complex concepts, and serving as a “study partner.” These results align with other studies where one of the main functions of AI for university students seems to be that of a private tutor or a study companion [23]. Many students highlighted its effectiveness in providing instant, pedagogical explanations, clarifying lecture material, and generating multiple-choice questions for self-assessment. Others used it to rephrase notes, structure responses, and identify key terms for further research. However, trust in generative AI varied, with students acknowledging the need to cross-check information with authoritative sources. One of the free-text answers describes the use of AI in order to “dumb down” a concept, something that might be hard to ask the lecturer to do. This use of AI is also something that is emphasized in a report by the Joint Information Systems Committee (United Kingdom) [24].

More than half of the students reported using generative AI to search for information, i.e., as a search engine. These results are consistent with those of another study from a Swedish university, where ChatGPT was found to be a commonly used search engine, preferred over other search engines, e.g. Google, because of the perceived effectiveness [25].

Interestingly, usage patterns differed based on baseline characteristics. Younger students (≤ 25 years) reported higher reliance on generative AI, particularly for exam preparation and conceptual explanations, whereas older students used it less frequently. Males reported more frequent AI use than females, particularly for text generation and summarization. Preclinical students engaged more with AI than their clinical counterparts, likely due to its effectiveness in theoretical learning rather than practical applications. Students generally viewed AI as a supplement rather than a replacement for other learning sources, comparing it to consulting a knowledgeable peer. However, self-reported data may not fully reflect AI usage, as university policies on AI-generated content differ, and some students may have hesitated to disclose their reliance on AI, particularly for submission tasks.

The increasing use of AI as a search engine and a personal tutor has highlighted the importance of evaluative judgement. Bearman et al. argue that it is necessary for students to be able to assess the quality of the output, that they will exercise evaluative judgement in the same way they need to when searching for information from, e.g., peers, teachers and literature. Otherwise students might uncritically rely on the outputs from ChatGPT. They also stress the importance of teachers helping them develop that skill [26].

Given its growing role in education, universities should establish clear guidelines for ethical generative AI use, ensuring it enhances rather than undermines critical thinking. Future research should explore whether AI-driven study methods improve knowledge retention and medical reasoning.

### Educational videos and audio podcasts

Medical school is a dynamic program. Whereas the preclinical phase focuses on basic science, such as anatomy, physiology and biochemistry, the following semesters have predominantly clinical content. In general, basic science topics may not be subject to as rapid changes that are often observed in case of more hands-on clinical areas, with important implications for which digital learning tools may be best suited during different phases. For example, educational videos, with potential to demonstrate practical procedures in a clear and pedagogical way, may be even more useful during clinical semesters [27, 28]. In particular when delivered in short formats (5–10 min), videos may be used throughout the day, in- and outside of the classroom, and even during clinical placements, enhancing flexibility and time management [28, 29]. However, a risk to consider, related to Youtube videos and videos in general, is the previously mentioned fact that students´ perception of learning is not necessarily correlated with their actual learning. Passively watching videos with famous and skilled lecturers can create such a positive feeling of learning that they prefer this modality over actively working with material in a way that may actually strengthen learning more [9]. Interestingly, although audio podcasts have gained enormous popularity throughout almost all clinical specialties, there appears to be a paucity of high-quality podcasts directed to medical students, at least in Sweden.

### Access and use of digital resources

Digital resources often entail costs. As the range of available AI tools expands, many of which are not free, issues of equity and the risk of unequal access based on ability to pay have been widely discussed [24, 30]. However, this concern was not strongly reflected among our respondents; only a few comments in the free-text responses mentioned cost. This may be attributed to the fact that Swedish higher education institutions typically provide broad access to digital resources. Institutional licenses are common, and several universities explicitly state in their AI guidelines that the institution is responsible for ensuring free and equitable access to AI tools used in teaching.

While more than half of the students reported use of digital tools being promoted by the institution, the main use of these resources seems to be part of students´ self-study activities, rather than the formal curriculum, recorded lectures being the main exception. Free-text responses indicate institutional differences. Further investigation would be needed to better understand how digital tools are incorporated into curricula and how equitable access is maintained.

### Methodological considerations

There are several limitations that should be considered. Although, to our knowledge, this is the only nationwide, and largest published survey study of digital resource use among medical students so far (*n* = 1766), the response rate was relatively low. It is possible that alternative recruitment strategies could have achieved higher response rates. We did consider some form of economic incentive to the class that produced the highest response rate, but decided not to do so, to avoid skewed driving forces behind study participation. Given the total response rate (20.2%), and the voluntary nature of participation, there may be an element of selection bias. Although the survey was promoted broadly, as an opportunity to support improvements in medical education, students with specific perspectives on, or more extensive experience with, digital resources may be overrepresented. Additionally, differences in response rates between universities may have introduced institutional bias, along with other confounding factors related to institutional variation. Moreover, 25/26 survey items were multiple choice questions; although the phrasing of questions and selection of response options were based on careful, iterative pilot testing and consensus discussions, some details and nuances may have been missed. Nevertheless, in general, very few selected “other” or “don´t know” alternatives, indicating that the provided response alternatives were adequate and sufficient to capture the most essential aspects. Some students pointed out that the use of digital resources varies between courses and semesters. This leads to a degree of uncertainty whether students answered certain questions from a perspective of “on average” or as a snapshot view. Important strengths of the study include the nationwide coverage, increasing generalizability, validated survey methodology and mixed methods data collection, which enabled not only large-scale quantitative analysis, but also qualitative, in-depth analysis. While our findings provide valuable insights into students’ perceptions and usage of digital tools, we acknowledge that perceptions of learning do not necessarily equate to actual learning gains or academic performance [9, 26, 31] This is a well-established limitation in educational research, particularly when relying on self-reported outcomes. Our study was not designed to assess direct academic outcomes (e.g., exam performance, long-term retention), which should be the subject of future investigations.

## Conclusions

Medical students frequently use, and perceive large positive learning effects and benefits from, digital learning resources. Several relevant background factors influence usage patterns. These data contribute to our understanding of medical students´ digital learning methods and techniques.

"Thank you for doing this! You truly have the chance to contribute to improving the medical school program (to bring the education 15 years forward in time… to where the students already are)."(participant #88).

This quote illustrates the discrepancy that often exists between students and faculty members. Our views on learning and the use of digital resources may differ. This key fact is further highlighted by multiple comments where students express a wish for more guidance from teachers. Based on both quantitative data and qualitative analysis there are calls to improve implementation and design of digital resources tailored to the curriculum, e.g., flashcards and high-quality videos developed by the educational institution itself, as well as calls to review and recommend third-party digital resources. If we as teachers fail to take responsibility to discuss these topics, show interest in the digital resources that are used, and at least to some extent guide the students, there is a risk that students will use learning techniques and resources that might lead to a feeling of learning rather than actual learning. Further mixed-methods pedagogical research is needed to advance our understanding of the utility of digital learning modalities for medical students.

## Supplementary Information

Supplementary Material 1. Supplemental Checklist. Checklist for Reporting of Survey Studies (CROSS).

Supplementary Material 2. Supplemental Fig. 1. Student distribution across semesters for the seven universities.

Supplementary Material 3. Supplemental Fig. 2. Differences in use of digital resources between the seven universities.

Supplementary Material 4. Supplemental Fig. 3. Illustration of a flashcard [AI-generated].

Supplementary Material 5. Supplemental Table 1. All digital resources reported in free-text format.

Supplementary Material 6. Supplemental Table 2. Overview of thematic analysis of free-text responses.

Supplementary Material 7. Supplemental Document 1. Survey of digital resource use among medical students in Sweden (translated English version).

Supplementary Material 8. Supplemental Document 2. Survey of digital resource use among medical students in Sweden (original Swedish version).

## Data Availability

The authors agree to make data supporting the results and analyses presented in the article available on request. Contact: Martin Flores Bjurström (martin.flores.bjurstrom@uu.se).
