# Supplementary figures and images for: Digital learning resource use among Swedish medical students: insights from a nationwide survey
Source: BMC Med Educ. 2025 Jun 11;25:849. doi: 10.1186/s12909-025-07446-7 (PMC12153187; doi:10.1186/s12909-025-07446-7)

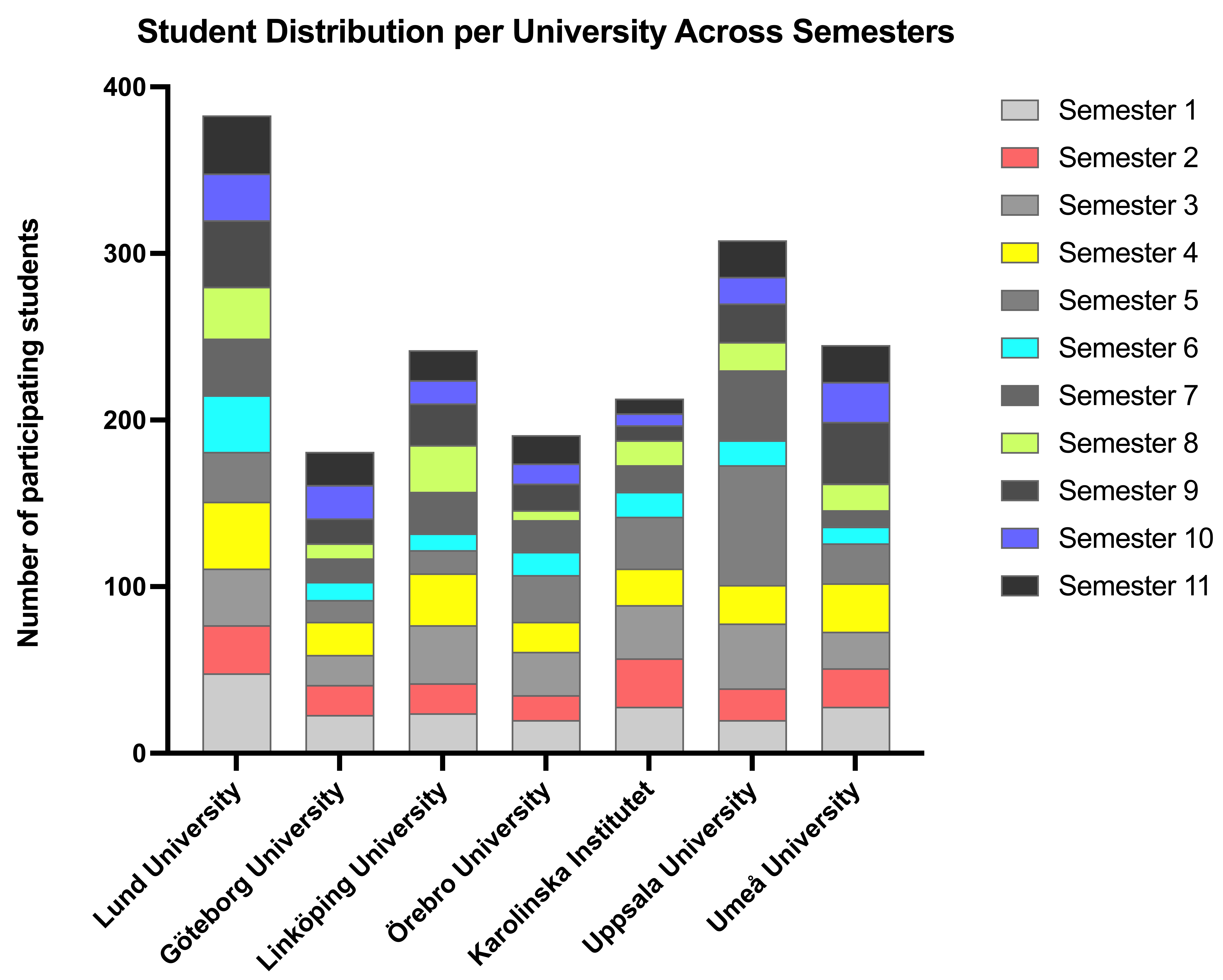

Supplement: Supplementary file 2 — Supplementary Material 2. Supplemental Fig. 1. Student distribution across semesters for the seven universities. [file 12909_2025_7446_MOESM2_ESM.jpg]

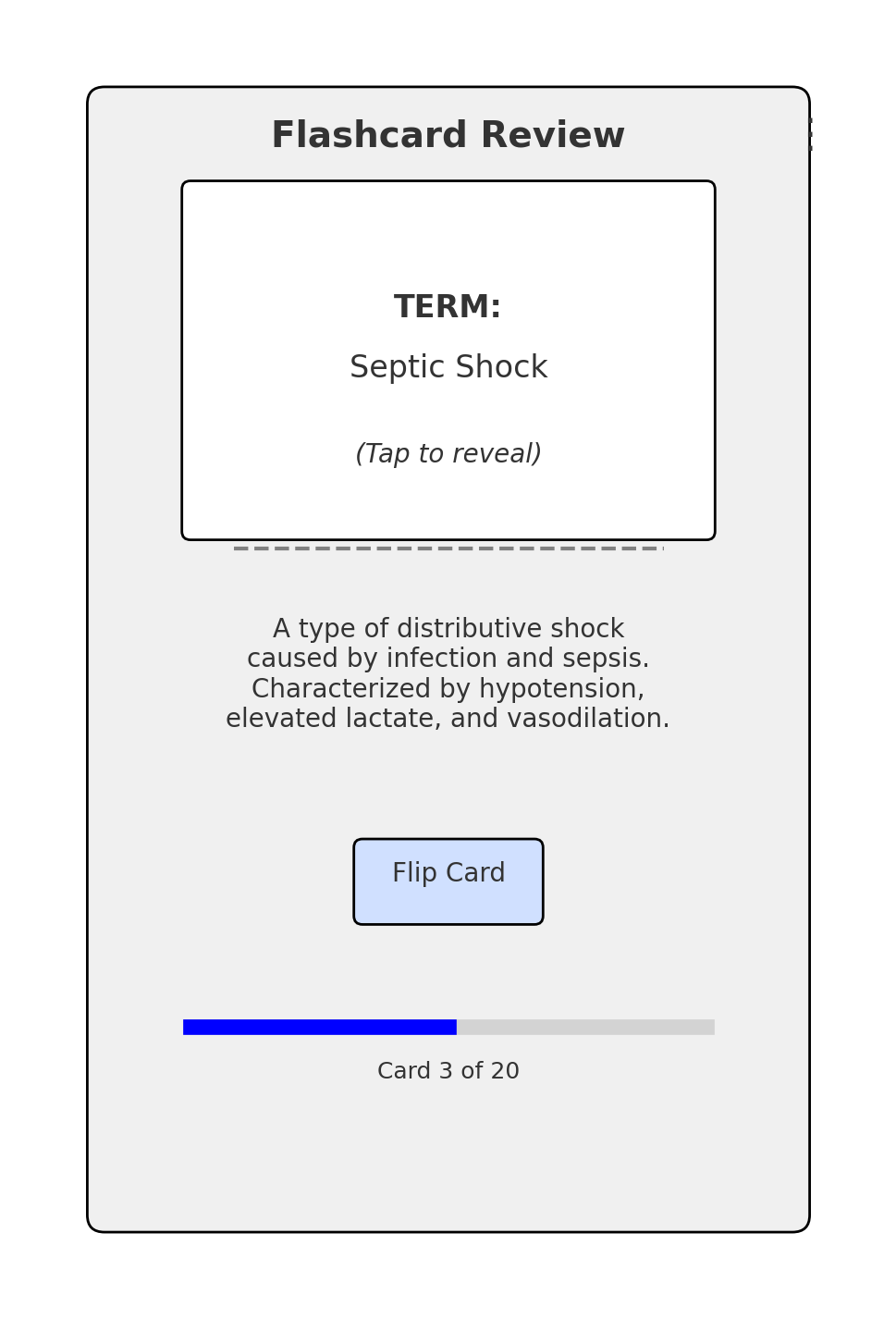

Supplement: Supplementary file 4 — Supplementary Material 4. Supplemental Fig. 3. Illustration of a flashcard [AI-generated]. [file 12909_2025_7446_MOESM4_ESM.png]
