# Supplementary material for: Digital learning resource use among Swedish medical students: insights from a nationwide survey
Source: BMC Med Educ. 2025 Jun 11;25:849. doi: 10.1186/s12909-025-07446-7 (PMC12153187; doi:10.1186/s12909-025-07446-7)
Supplement: Supplementary file 5 — Supplementary Material 5. Supplemental Table 1. All digital resources reported in free-text format. [file 12909_2025_7446_MOESM5_ESM.docx]

# Supplemental Table 1 – Complete list of reported digital resources.

| Name | Short description | Source/developer | Percentage of students mentioning* |
| --- | --- | --- | --- |
| Flashcard programs | | | |
| Anki | Software program for creating flashcards and using premade flashcards. | Open source | 48.5 |
| Quizlet | Software program for constructing and sharing flashcards. | Quizlet Inc | 4.2 |
| Flashcards unspec. |  |  | 7.5 |
| AI | | | |
| Chat GPT | Generative AI tool based on a large language model (LLM). | OpenAI | 14.9 |
| AI unspec. |  |  | 1.8 |
| Youtube channels (and other videos) | | | |
| Ninja Nerd | Science and medicine lectures on broad topics | Ninja Nerd | 17.0 |
| Youtube unspec. |  |  | 28.7 |
| Videos unspec. |  |  | 1.5 |
| Study platforms | | | |
| University digital study platform, e.g. Canvas | Learning management system, provided by the university | Various | 8.8 |
| Hypocampus | Digital study platform for medical students, built on pedagogical and cognitive science | Hypocampus AB | 26.6 |
| Osmosis | American platform with online videos, articles,  and questions | Elsevier | 18.9 |
| Complete Anatomy | An anatomy software with 3D anatomy tools and materials | Elsevier | 3.3 |
| Student organized platforms for sharing study material | Various material, e.g. study tips, PBL cases, premade flashcards, student assignments | Previous and current medical students | 7.2 |
| Webpages | | | |
| Internetmedicin | Swedish webpage with overviews on diagnostics and treatment of different diseases | Medical experts | 16.4 |
| Wikipedia | Online encyclopaedia, created and edited by volunteers | Wikimedia Foundation | 1.7 |
| Databases and search engines | | | |
| PubMed | Database for biomedical and life sciences literature | U.S. National Library of Medicine (NLM) | 2.4 |
| ClinicalKey | Clinical search engine with books, medical journals, images, videos etc | Elsevier | 7.6 |
| Google | Search engine | Alphabet (holding company) | 3.9 |
| FASS | Quality-assured pharmaceuticals information on all approved medicines in Sweden. | LIF, the research-based pharmaceutical industry in Sweden | 1.2 |
| Visible body | An anatomy database with several tools for creating and working with anatomical views | Visible body | 2.3 |
| Medibas | Evidence based knowledge, focusing on primary care in Sweden | Bonnier Healthcare Sweden | 1.5 |
| Workspace and notebook applications | | | |
| OneNote | Digital notebook | Microsoft | 1.5 |
| Trello and Notion | Project workspace apps | Atlassian and Notion Labs, Inc. | 2.1 |
| Misc. resources | | | |
| Recorded lectures |  |  | 14.4 |
| Handouts |  |  | 2.3 |
| Notes and previous exams from senior students |  |  | 13.7 |
| E-books |  |  | 8.6 |
| Digital articles |  |  | 1.9 |
| Guidelines online |  |  | 1.2 |

*The percentage refers to the proportion of participants mentioning the digital resource in response to survey question #26, i.e., “Which are the three digital resources that have helped you the most during the medical program?” In total, 1426 (80.7%) responded to question #26.
