# Supplementary material for: Digital learning resource use among Swedish medical students: insights from a nationwide survey
Source: BMC Med Educ. 2025 Jun 11;25:849. doi: 10.1186/s12909-025-07446-7 (PMC12153187; doi:10.1186/s12909-025-07446-7)
Supplement: Supplementary file 6 — Supplementary Material 6. Supplemental Table 2. Overview of thematic analysis of free-text responses. [file 12909_2025_7446_MOESM6_ESM.pptx]

## Slide 1
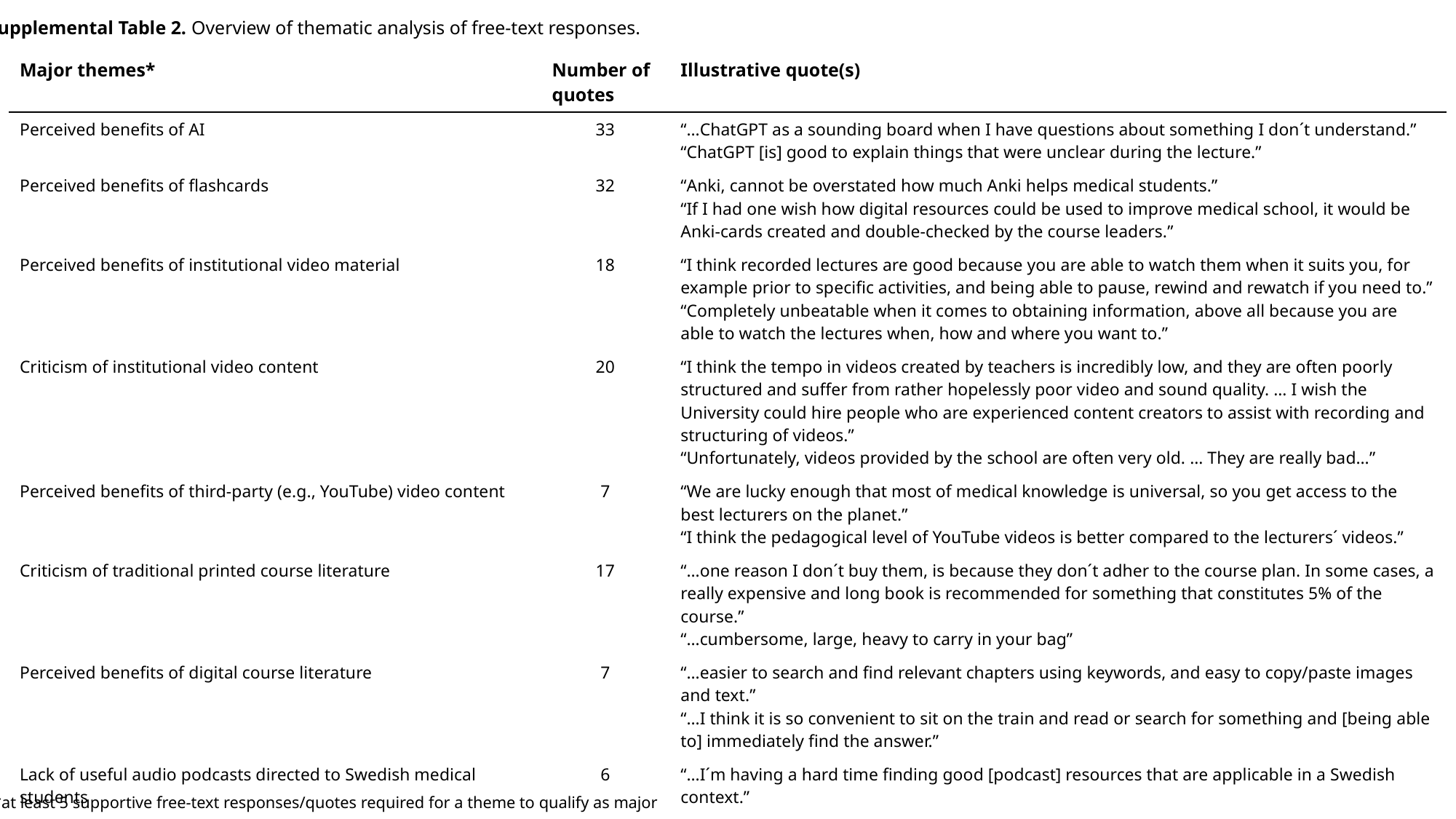

Supplemental Table 2. Overview of thematic analysis of free-text responses.
| Major themes\* | Number of quotes | Illustrative quote(s) |
| --- | --- | --- |
| Perceived benefits of AI | 33 | “…ChatGPT as a sounding board when I have questions about something I don´t understand.” “ChatGPT [is] good to explain things that were unclear during the lecture.” |
| Perceived benefits of flashcards | 32 | “Anki, cannot be overstated how much Anki helps medical students.” “If I had one wish how digital resources could be used to improve medical school, it would be Anki-cards created and double-checked by the course leaders.” |
| Perceived benefits of institutional video material | 18 | “I think recorded lectures are good because you are able to watch them when it suits you, for example prior to specific activities, and being able to pause, rewind and rewatch if you need to.” “Completely unbeatable when it comes to obtaining information, above all because you are able to watch the lectures when, how and where you want to.” |
| Criticism of institutional video content | 20 | “I think the tempo in videos created by teachers is incredibly low, and they are often poorly structured and suffer from rather hopelessly poor video and sound quality. … I wish the University could hire people who are experienced content creators to assist with recording and structuring of videos.” “Unfortunately, videos provided by the school are often very old. … They are really bad…” |
| Perceived benefits of third-party (e.g., YouTube) video content | 7 | “We are lucky enough that most of medical knowledge is universal, so you get access to the best lecturers on the planet.” “I think the pedagogical level of YouTube videos is better compared to the lecturers´ videos.” |
| Criticism of traditional printed course literature | 17 | “…one reason I don´t buy them, is because they don´t adher to the course plan. In some cases, a really expensive and long book is recommended for something that constitutes 5% of the course.” “…cumbersome, large, heavy to carry in your bag” |
| Perceived benefits of digital course literature | 7 | “…easier to search and find relevant chapters using keywords, and easy to copy/paste images and text.” “…I think it is so convenient to sit on the train and read or search for something and [being able to] immediately find the answer.” |
| Lack of useful audio podcasts directed to Swedish medical students | 6 | “…I´m having a hard time finding good [podcast] resources that are applicable in a Swedish context.” |
| Differences in use of digital resources over the course of the education | 13 | “I have used different resources during different stages [of the program]. Preclinical, more videos, clinical, more internetmedicin[.se].” “More notes from senior students during preclinical [semesters] and Hypocampus during clinical [semesters].” |
*at least 5 supportive free-text responses/quotes required for a theme to qualify as major
