# Supplementary material for: Digital learning resource use among Swedish medical students: insights from a nationwide survey
Source: BMC Med Educ. 2025 Jun 11;25:849. doi: 10.1186/s12909-025-07446-7 (PMC12153187; doi:10.1186/s12909-025-07446-7)
Supplement: Supplementary file 7 — Supplementary Material 7. Supplemental Document 1. Survey of digital resource use among medical students in Sweden (translated English version). [file 12909_2025_7446_MOESM7_ESM.docx]

PREAMBLE:

Dear Medical Student,

We are a group of doctors, researchers and educators from three different universities (Lund University, Karolinska Institutet, Uppsala University) who are currently conducting a survey that focuses on digital resources, i.e., learning methods that you can decide where and when you want to use.

We would be extremely grateful if you had the opportunity to answer the questions included in this survey (takes about 5 – 10 minutes), so that we can better understand the learning methods medical students use in 2024. Regardless of which semester you are attending, we would like to ask you to think about the education so far in general, i.e., not just focus on the course or courses you are currently taking.

Your answers are valuable – our hope is that the results can be used to improve medical education and make studies easier for you as a medical student!

1. At which university are you studying the medical program?

□ Lund University

□ University of Gothenburg / Sahlgrenska Academy

□ Linköping University

□ Örebro University

□ Karolinska Institutet

□ Uppsala University

□ Umeå University

2. What semester are you currently attending?

Drop-down list with options 1 - 12

3. How old are you?

□ <20 years

□ 20 - 25 years old

□ 26 - 30 years

□ 31 - 35 years old

□ 36 - 40 years

□ >40 years

4. Sex

□ woman

□ man

□ other

□ do not want to disclose

5. What digital resources do you use in connection with your studies in the medical program?

□ videos

□ podcasts

□ flashcards

□ groups via social media (e.g., Facebook groups)

□ Generative AI (e.g., chat robots like ChatGPT)

□ more senior students' digital notes/summaries (via e.g. Google Drive, Dropbox, Studocu)

□ the university digital study platform

□ external digital study platform (e.g. Hypocampus, Osmosis)

□ digital books

□ digital articles

□ other

□ don't know

6. How often do you use different digital resources in your medical program?

Videos, podcasts, flashcards, groups via social media, generative AI, more senior students' digital notes/summaries, university digital study platform, external digital study platform, digital books, digital articles, other

□ never

□ a few times

□ 1 - 2 times/month

□ 1 time/week

□ a few times/week

□ daily

□ don't know

7. How do you usually find the digital resources you use?

□ tip from medical students attending the same semester

□ tip from teaching assistants/medical students attending higher semesters

□ tip from course management

□ tip/advertising in connection with the use of other digital resources

□ via online search engine

□ other ways

□ don't know

8. Is the use of digital resources encouraged by the course management, e.g., via tip in the study platform?

□ never

□ rarely

□ sometimes

□ often

□ don't know

9. To what extent is your use of digital resources affected by any encouragement from the course management?

□ not at all

□ to a low degree

□ to a moderate degree

□ to a large extent

□ don't know

10. In what situations do you most often use digital resources? (up to three options can be selected)

□ in parallel with other educational activities in the classroom/lecture hall

□ during individual studies

□ during group studies

□ during the day when I rest

□ during the day in connection with household work

□ in connection with commuting to and from studies

□ in connection with training

□ in bed before going to sleep

□ other situation

□ don't know

11. What advantages do you perceive using digital resources as learning methods? (check all that apply)

□ accessibility, can decide when and where

□ the opportunity to do other things in the meantime

□ the opportunity to pause, repeat

□ up-to-date, relevant content

□ more effective learning compared to other more traditional learning methods

□ I don't see any special advantages

□ don't know

12. What disadvantages or risks do you perceive using digital resources as learning methods? (check all that apply)

□ easy to get distracted

□ not always reliable content

□ hard to find the content I'm looking for

□ ineffective learning compared to other more traditional learning methods

□ too many ad breaks

□ difficult to separate study and leisure time

□ I don't see any specific disadvantages or risks

□ don't know

13. What benefits do you see with educational videos? (check all that apply)

□ accessibility, can decide when and where

□ adds dimensions that are difficult to capture through other forms of teaching

□ the ability to pause, replay

□ effective way to quickly gain concentrated knowledge

□ preparation for upcoming activity/teaching in the classroom

□ more effective learning compared to other more traditional forms of teaching

□ I don't see any specific advantages

□ I don't use videos for learning

□ don't know

14. What disadvantages do you see with teaching videos? (check all that apply)

□ easy to get distracted

□ subpar quality – ends the video prematurely

□ hard to find videos that show what I want to learn

□ difficult to determine whether the content of the video is applicable in a Swedish context

□ not reliable information

□ too many ad breaks

□ I don't see any specific disadvantages

□ I don't use videos

□ don't know

15. What are the top three reasons why you don't watch an entire educational video, i.e., stop watching the video prematurely?

□ the video is too long

□ the pace is too slow

□ video doesn't meet my learning needs

□ not a sufficiently focused message

□ not engaging enough lecturers

□ content doesn't match the title

□ poor sound quality

□ poor image quality

□ the training does not allocate enough time to watch recommended video material

□ don't know

16. If you exercise while listening to podcasts related to the medical program – how would you estimate the impact on your learning?

□ no impact

□ much worse effect

□ poorer effect

□ some positive effect

□ very positive effect

□ I don't listen to podcasts or exercise

□ don't know

17. To what extent do you think flashcards help to develop and preserve your theoretical knowledge?

□ none

□ very small

□ quite small

□ moderate

□ large

□ very large

□ don't know

□ I don't use flashcards

18. How do you get access to digital flashcards? (check all that apply)

□ create flashcards myself

□ create with the help of programs that generate flashcards based on text/notes

□ receive from medical students who attend the same semester

□ acquire from more senior medical students

□ freely available flashcards online

□ buy from other students

□ buy online

□ don't know

□ I don't use flashcards

19. Why do you use generative AI (e.g., ChatGPT)?

□ adjust and improve the language of my text

□ translate texts

□ summarize texts

□ seek information/get answers to questions

□ get ideas and ideas to get started with tasks

□ get feedback on texts I´ve written

□ other reason

□ don't know

□ I don't use generative AI (if this option automatically move to question 21)

20. How often do you use generative AI (e.g. chatbots like ChatGPT) in connection with assignments? Note that this question examines usage patterns and thus DOES NOT aim to map the unauthorized use of generative AI.

□ never

□ rarely

□ sometimes

□ often

□ always

□ don't know

21. How often do you use (borrow or buy) the recommended course literature?

□ for each course

□ for most courses

□ for individual courses

□ never

□ don't know

22. If you don't use the course literature, what is the main reason?

□ expensive

□ have not been able to borrow the book

□ it is not necessary

□ too hard to read

□ learn better with other materials/methods

□ always use course literature

□ don't know

23. Do you have work experience and/or experience of other higher education studies prior to starting the medical program? (check all that apply)

□ no

□ yes, higher education single course(s)

□ yes, higher education full program

□ yes, vocational education

□ yes, residential college for adult education

□ yes, military service

□ yes, work experience <5 years

□ yes, work experience ≥5 years

24. Do you do extracurricular work during semesters?

□ no

□ yes, once in a while

□ yes, 5 – 10 hours per week

□ yes, 11 – 20 hours per week

□ yes, >20 hours per week

25. Do you have children?

□ yes, living at home

□ yes, not living at home

□ no

□ do not want to disclose

26. Which are the three digital resources that have helped you the most during the medical program? If there is anything else you want to add, e.g., something we might have missed to ask about, we would be very grateful if you could tell us here.

[free text]
