# Supplementary material for: Digital learning resource use among Swedish medical students: insights from a nationwide survey
Source: BMC Med Educ. 2025 Jun 11;25:849. doi: 10.1186/s12909-025-07446-7 (PMC12153187; doi:10.1186/s12909-025-07446-7)
Supplement: Supplementary file 8 — Supplementary Material 8. Supplemental Document 2. Survey of digital resource use among medical students in Sweden (original Swedish version). [file 12909_2025_7446_MOESM8_ESM.docx]

INGRESS:

Bästa läkarstudent,

Vi är en grupp läkare, forskare och pedagoger från tre olika lärosäten (Lunds universitet, Karolinska institutet, Uppsala universitet) som just nu genomför en undersökning som fokuserar på digitala resurser, dvs lärandemetoder som du själv kan bestämma var och när du vill använda.

Vi vore oerhört tacksamma om du har möjlighet att besvara de frågor som ingår i denna enkät (tar ca 5 – 10 minuter), så att vi bättre kan förstå de lärandemetoder läkarstudenter använder sig av 2024. Oavsett vilken termin du går på, vill vi be dig att tänka på utbildningen hittills överlag, dvs fokusera inte bara på den kurs eller de kurser du för tillfället går.

Dina svar är värdefulla – vår förhoppning är att resultaten kan användas för att förbättra läkarutbildningen och underlätta studierna för dig som läkarstudent!

1. Vid vilket lärosäte gör du din läkarutbildning?

□ Lunds universitet

□ Göteborgs universitet / Sahlgrenska akademin

□ Linköpings universitet

□ Örebro universitet

□ Karolinska institutet

□ Uppsala universitet

□ Umeå universitet

2. Vilken termin går du för närvarande?

Rullista med alternativ 1 - 12

3. Hur gammal är du?

□ <20 år

□ 20 - 25 år

□ 26 - 30 år

□ 31 - 35 år

□ 36 - 40 år

□ >40 år

4. Kön

□ kvinna

□ man

□ annat

□ vill inte uppge

5. Vilka digitala resurser använder du dig av i samband med dina studier på läkarutbildningen?

□ videor

□ podcasts

□ flashcards

□ grupper via sociala media (ex. facebookgrupper)

□ generativ AI (ex. chatrobotar som ChatGPT)

□ äldrekursares digitala anteckningar/sammanfattningar (via ex. Google Drive, Dropbox, Studocu)

□ universitetets digitala studieplattform

□ extern digital studieplattform (ex. Hypocampus, Osmosis)

□ digitala böcker

□ digitala artiklar

□ annat

□ vet ej

6. Hur ofta använder du olika digitala resurser för din läkarutbildning?

Videor, podcasts, flashcards, grupper via sociala media, generativ AI, äldrekursares digitala anteckningar/sammanfattningar, universitetets digitala studieplattform, extern digital studieplattform, digitala böcker, digitala artiklar, annat

□ aldrig

□ enstaka gång

□ 1 - 2 gånger/månad

□ 1 gång/vecka

□ några gånger per vecka

□ dagligen

□ vet ej

7. Hur hittar du vanligen de digitala resurser som du använder dig av?

□ tips från läkarstudenter som går samma termin

□ tips från amanuenser / läkarstudenter som går högre termin

□ tips från kursledning

□ tips/reklam i samband med användning av andra digitala resurser

□ via sökmotor online

□ annat sätt

□ vet ej

8. Uppmuntras användning av digitala resurser från kursledningen, ex. via tips i studieplattform?

□ aldrig

□ sällan

□ ibland

□ ofta

□ vet ej

9. I vilken grad påverkas ditt användande av digitala resurser av ev. uppmuntran från kursledningen?

□ inte alls

□ i låg grad

□ i måttlig grad

□ i hög grad

□ vet ej

10. I vilka situationer använder du dig oftast av digitala resurser? (upp till tre alternativ kan väljas)

□ parallellt med annan pedagogisk aktivitet i klassrum/föreläsningssal

□ under individuella studier

□ under studier i grupp

□ under dagen när jag vilar

□ under dagen i samband med hushållsarbete

□ i samband med pendling till och från studier

□ i samband med träning

□ i sängen inför nattsömn

□ annan situation

□ vet ej

11. Vilka fördelar ser du med användande av digitala resurser som lärandemetoder? (kryssa i alla som gäller)

□ tillgänglighet, kan själv bestämma när och var

□ möjlighet att göra annat under tiden

□ möjlighet att pausa, repetera

□ uppdaterat, relevant innehåll

□ effektivare lärande jämfört med andra mer traditionella lärandemetoder

□ jag ser inga speciella fördelar

□ vet ej

12. Vilka nackdelar eller risker ser du med användande av digitala resurser som lärandemetoder? (kryssa i alla som gäller)

□ lätt att bli distraherad

□ inte alltid tillförlitligt innehåll

□ svårt att hitta det innehåll jag söker

□ ineffektivt lärande jämfört med andra mer traditionella lärandemetoder

□ för många reklamavbrott

□ svårt att sätta gräns mellan studier och fritid

□ jag ser inga speciella nackdelar eller risker

□ vet ej

13. Vilka fördelar ser du med undervisningsvideor? (kryssa i alla som gäller)

□ tillgänglighet, kan själv bestämma när och var

□ bidrar med dimensioner som är svåra att fånga med andra undervisningsformer

□ möjlighet att pausa, spela upp video på nytt

□ effektivt sätt att snabbt få koncentrerad kunskap

□ förberedelse för kommande aktivitet/undervisning i klassrum

□ effektivare lärande jämfört med andra mer traditionella undervisningsformer

□ jag ser inga speciella fördelar

□ jag använder ej videor i samband med inlärning

□ vet ej

14. Vilka nackdelar ser du med undervisningsvideor? (kryssa i alla som gäller)

□ lätt att bli distraherad

□ undermålig kvalitet – avslutar videon i förtid

□ svårt att hitta video som visar det jag vill lära mig

□ svårt att avgöra om videons innehåll är applicerbart i en svensk kontext

□ inte tillförlitlig information

□ för många reklamavbrott

□ jag ser inga speciella nackdelar

□ jag använder ej videor

□ vet ej

15. Vilka är de tre främsta anledningarna till att du inte ser en hel undervisningsvideo, dvs avbryter videon i förtid?

□ videon är för lång

□ tempot är för lågt

□ videon tillgodoser inte mina lärandebehov

□ inte tillräckligt fokuserat budskap

□ inte tillräckligt engagerande föreläsare

□ innehållet överensstämmer inte med titeln

□ bristfällig ljudkvalitet

□ bristfällig bildkvalitet

□ utbildningen avsätter inte tillräckligt med tid för att se rekommenderat videomaterial

□ vet ej

16. Om du tränar samtidigt som du lyssnar på podcasts relaterade till läkarutbildningen – hur skulle du uppskatta effekten på din inlärning?

□ ingen påverkan

□ mycket sämre effekt

□ sämre effekt

□ viss positiv effekt

□ mycket positiv effekt

□ jag lyssnar ej på podcasts eller tränar ej

□ vet ej

17. I vilken omfattning tycker du att flashcards bidrar till att utveckla och bevara dina teoretiska kunskaper?

□ ingen

□ mycket liten

□ ganska liten

□ måttlig

□ stor

□ mycket stor

□ vet ej

□ jag använder inte flashcards

18. Hur får du tillgång till digitala flashcards? (kryssa i alla som gäller)

□ skapar flashcards själv

□ skapar med hjälp av program som baserat på text/kompendier genererar flashcards

□ får från läkarstudenter som går samma termin

□ får från läkarstudenter som går högre termin

□ fritt tillgängliga flashcards online

□ köper av andra studenter

□ köper online

□ vet ej

□ jag använder inte flashcards

19. Varför använder du dig av generativ AI (ex. chatrobotar som ChatGPT)?

□ justera och förbättra språket i min text

□ översätta texter

□ sammanfatta texter

□ söka information/få svar på frågor

□ få uppslag och idéer för att komma igång med uppgifter

□ få återkoppling på texter jag skrivit

□ annat

□ vet ej

□ jag använder inte generativ AI (om detta alternativ automatiskt vidare till fråga 21)

20. Hur ofta använder du dig av generativ AI (ex. chatrobotar som ChatGPT) i samband med inlämningsuppgifter? Observera att denna fråga undersöker användningsmönster och alltså EJ syftar till att kartlägga otillåtet användande av generativ AI.

□ aldrig

□ sällan

□ ibland

□ ofta

□ alltid

□ vet ej

21. Hur ofta använder (lånar eller köper) du den rekommenderade kurslitteraturen?

□ på varje kurs

□ på de flesta kurser

□ på enstaka kurser

□ aldrig

□ vet ej

22. Om du inte använder kurslitteraturen, vad är den främsta anledningen till det?

□ dyrt

□ har inte kunnat låna boken

□ behövs inte

□ för svår att läsa

□ lär mig bättre med annat material/andra metoder

□ använder alltid kurslitteratur

□ vet ej

23. Har du arbetslivserfarenhet och/eller genomgått andra postgymnasiala studier före det att du påbörjade läkarutbildningen? (kryssa i alla som gäller)

□ nej

□ ja, högskoleutbildning enstaka kurs(er)

□ ja, högskoleutbildning helt program

□ ja, yrkesutbildning

□ ja, folkhögskoleutbildning

□ ja, militärtjänstgöring

□ ja, arbetslivserfarenhet <5 år

□ ja, arbetslivserfarenhet ≥5 år

24. Arbetar du vid sidan av studierna under terminstid?

□ nej

□ ja, någon gång då och då

□ ja, 5 – 10 timmar per vecka

□ ja, 11 – 20 timmar per vecka

□ ja, >20 timmar per vecka

25. Har du barn?

□ ja, hemmaboende

□ ja, ej hemmaboende

□ nej

□ vill inte uppge

26. Vilka är de tre digitala resurser som har hjälpt dig mest under läkarutbildningen? Är det något annat du vill lägga till, ex. något vi missat att fråga om, så vore vi väldigt tacksamma om du berättar här.

[fritext]
